# Music therapy, neural processing, and craving reduction: an RCT protocol for a mixed methods feasibility study in a Community Substance Misuse Treatment Service

**DOI:** 10.1186/s13722-023-00385-y

**Published:** 2023-05-27

**Authors:** Jörg Fachner, Clemens Maidhof, Daniel Murtagh, Devon De Silva, Filippo Pasqualitto, Paul Fernie, Francesca Panin, Andrew Michell, Leonardo Muller-Rodriguez, Helen Odell-Miller

**Affiliations:** 1 Cambridge Institute for Music Therapy Research, Anglia Ruskin University, Cambridge, UK; 2 Innovation & Research Unit, Westminster Drug Project (WDP), London, UK; 3 School of Life Sciences, Faculty of Science and Engineering, Anglia Ruskin University, Cambridge, UK; 4 Clinical Neurophysiology, Addenbrooke’s Hospital, Cambridge, UK

**Keywords:** Substance use disorder, Music therapy, Community treatment service, Feasibility, Randomized controlled trial, Mental health, Neural processing, EEG, Mixed methods

## Abstract

**Background:**

Music therapy has been shown to be effective for multiple clinical endpoints associated with substance use disorder such as craving reduction, emotion regulation, depression, and anxiety, but there are a lack of studies investigating those effects in UK Community Substance Misuse Treatment Services (CSMTSs). Furthermore, there is a demand for identifying music therapy mechanisms of change and related brain processes for substance use disorder treatment. The present study aims to evaluate the feasibility and acceptability of music therapy and a pre-test, post-test, and in-session measurement battery in a CSMTS.

**Methods:**

Fifteen participants, from a community service based in London, will take part in a mixed-methods non-blind randomized-controlled trial. Ten participants will receive six-weekly sessions of music therapy in addition to the standard treatment offered by the CSMTS—five of them will receive individual music therapy and five of them will receive group music therapy—while a further five participants will act as a control group receiving standard treatment only.

Satisfaction and acceptability will be evaluated in focus groups with service users and staff members following the final treatment session. Moreover, attendance and completion rates will be monitored throughout the intervention. Subjective and behavioral indexes will be assessed before and after the interventions to explore the effects of music therapy on craving, substance use, symptoms of depression and anxiety, inhibitory control, and will be correlated with associated neurophysiological signatures. In-session analysis of two individual music therapy sessions will serve to explore how music and emotion are processed in the brain within the therapy. The data collected at each step will be included in an intention-to-treat analysis basis.

**Discussion:**

This study will provide a first report on the feasibility of music therapy as an intervention for participants with substance use disorder engaged within a community service. It will also provide valuable information regarding the implementation of a multifaceted methodology that includes neurophysiological, questionnaire-based, and behavioral assessments in this cohort. Notwithstanding the limitation of a small sample size, the present study will provide novel preliminary data regarding neurophysiological outcomes in participants with substance use disorder that received music therapy.

*Trial Registration:* ClinicalTrails.gov, NCT0518061, Registered 6 January 2022, https://clinicaltrials.gov/ct2/show/NCT05180617

## Background

It is estimated that over 5% of the United Kingdom population suffers from Substance Use Disorder (SUD) which is directly (i.e., substance overdoses) or indirectly (i.e., substance use as a risk factor for premature death) considered responsible for over 4,000 deaths each year [1]. Among the people who accessed support with substances via adult drug and alcohol services from the National Health Service (NHS) and non-NHS providers in the UK between 2019 and 2020, only 3% of them received treatment in an inpatient setting and 2% of them in a residential setting [2], with numbers declining further for the period 2020 and 2021 (i.e., only 3% received treatment in an inpatient setting and 1% in a residential setting) [3]. In contrast, Community Substance Misuse Treatment Services (CSMTSs), inpatient detoxification, residential rehabilitation or private counseling see a much larger number of clients across the country. The generalization and application of the evidence base of Music therapy treatment [4], which is largely based on inpatient settings, remains challenging.

Despite the growing interest in using music therapy (MT) in SUD treatment [5–7], there is similarly a marked absence of studies that include participants from CSMTSs, forming a gap in the literature that needs to be addressed [4, 6, 7]. The current protocol aims at outlining the steps for covering this gap.

SUD has been defined as a chronic, and often relapsing disorder [8–10] targeting multiple interacting neural circuits in the brain [8, 9, 11] and associated with a cluster of cognitive, physiological, and behavioral symptoms indicating that the individual continues to use the substance despite adverse consequences such as craving, anxiety and depressive symptoms [12]. Research on the neuroscience of music showed that processing of pleasing musical stimuli is associated with overlapping patterns of neural activations induced by euphoretic psychoactive drugs [13, 14], suggesting how MT may modulate dopaminergic-dependent neural circuits and work on emotional reactions. This might help clients to overcome the compulsive characteristics of this condition and its state-dependent memories [15]. A recent Cochrane systematic review identified MT as a valuable SUD add-on treatment to standard care [4]. MT is defined as a clinical intervention delivered by an accredited music therapist adopting music and its elements as a therapeutic tool to accomplish individualized goals within a therapeutic alliance and facilitate positive changes in clients’ emotion regulation, motivation, and social engagement [4, 16–18]. The physiological effects of MT can be examined non-invasively through neuroscience methods, such as electroencephalography (EEG) to measure brain activity [19, 20]. Throughout MT sessions, important moments that represent personal changes accompanied by shared emotions and mutual understanding, can be identified [21, 22]. Recent research suggested that during moments of therapeutic interest (e.g., emotionally relevant moments) there exists increased synchronization of brain activity between the music therapist and the participant [22]. These moments can be further examined through hyperscanning technology [23, 24] allowing for the simultaneous recording of the brain activity of both the music therapist and the participant [21, 22]. Combining EEG-hyperscanning with audio–video (AV) recording of therapy sessions represents an innovative approach to tracking the temporal dynamics of event-related peaks related to emotional processing during therapy. The analysis of these data would build on the existing research regarding interacting brain synchronization [22, 24] and further improve our understanding of brain activity during possible emotionally challenging moments in the therapy, such as the experience of a craving state.

Craving is considered a key symptom for SUD, maintaining the pathological vicious circle of this condition [11, 25] and manifesting itself as an “intense desire or urge for the drug” [12]. Furthermore, anxiety and depression are two common conditions in individuals using substances [12] and MT has shown efficacy in reducing depressive [16, 17] and anxiety symptoms [16, 18] in individuals not suffering from SUD. Studies including SUD populations suggested that mental health clinical endpoints improved in those participants after receiving MT: beneficial effects have been pointed out for depressive symptoms [26, 27], anxiety symptoms [26, 28, 29], negative emotions (i.e., anger) [26] and craving [30–32]. The importance of measuring mental health indexes in MT settings for SUD and assessing brain data to explore the underlying mechanisms of a potential music-therapeutic success have been recently emphasized [4]. The reduction of craving, depression and anxiety are primary outcomes for participants receiving MT in addition to standard care, when compared to participants receiving standard care alone; especially if the MT intervention lasted from 1 to 3 months [4]. However, despite empirical evidence suggesting that MT is effective for SUD individuals, outcomes from studies using heterogeneous methodologies and MT intervention types are not always consistent [6]. Likewise, despite reported improvements in several mental health clinical endpoints, the mechanism of therapeutic change underlying the effects of MT for these variables is yet to be identified [4] and future randomized control trials adopting neuroscience research methods may provide insights not only on the efficacy of MT on relevant symptoms associated with SUD (e.g., depression, anxiety and craving) but also on the pre-post MT changes in brain activity that is hypothesized to underpin those conditions.

A recent Cochrane systematic review recommends that research on MT for SUD individuals should (I) include a broader diversity of treatment settings while decreasing heterogeneity within and between studies, (II) consider the associations between mental health and SUD, (III) evaluate the impact of length of treatment, (IV) assess outcomes at medium- and long-term follow-up, (V) match MT intervention to the phase of SUD treatment, and (VI) determine mechanisms of therapeutic change [4]. The study reported here will address some of those recommendations (diversity, mental health, treatment length) to determine the feasibility of implementing MT as an intervention for participants with SUD that are engaged within CSMTSs, a hitherto underrepresented cohort. In addition, the present study will test the feasibility of applying a pretest, posttest, and in-session measurement battery within a CSMTS to explore the efficacy and underlying mechanisms of MT change. Indeed, the novel nature of the current research project is the analysis of mechanisms of therapeutic change from two angles: (I) through the combination of subjective self-report measurements and objective EEG assessment comparing pre/post intervention changes and (II) through the study of MT session delivery and creative process analysis. The latter will also be supported by utilizing Electronic Music Production Instruments (EMPI) during therapy. EMPIs provide significant benefits to the analysis of musical interactions through incorporated multi-track recording functionality, enabling the separation of participant and therapist instrument play and affording detailed analysis of the musical mechanisms at work [33]. Thus, the project has the potential to identify an intervention that may enhance the mental wellbeing and recovery journey for individuals experiencing SUD and enhance the care offered by CSMTSs in the long term.

## Methods

### Study aims and specific objectives

The main aim of this study is to test the feasibility of implementing individual music therapy (IMT) and group music therapy (GMT) as an intervention for service users (SUs) experiencing symptoms of depression and/or anxiety and engaged with CSMTSs. In order to approach the primary aim, our objectives are (I) to measure qualitative data on SU satisfaction with the MT program, (II) to measure retention in treatment and (III) to collect their feedback over the course of the treatment. The secondary aims of this project are to examine the feasibility of applying a pre-test, posttest, and in-session assessment battery as well as to measure the effects of MT (GMT and IMT) on mental health and the neural processing associated with it. We aim to explore the effects of MT in addition to standard treatment (ST) versus standard treatment alone. Further secondary aims concern the exploration of features of the therapeutic relationship and the role of music technology in the delivery, recording, and analysis of music therapy sessions. In order to approach the secondary aims, our objectives are: (I) To measure the effects of MT in addition to ST on the following treatment outcomes in comparison to ST alone: (a) substance use, (b) physical, mental wellbeing and quality of life, (c) retention in treatment, (d) engagement with treatment, (e) completion rate, (f) emotional processing, (g) psychological symptomatology. (II) To measure reduction in craving intensity after single MT sessions. (III) To compare the effect of MT in addition to ST versus ST alone on craving, inhibitory control, depression, anxiety and the respective associated neural signatures. (IV) To explore the perceived therapeutic alliance in IMT sessions. (V) To explore the neural dynamics of emotional processing during IMT sessions. (VI) To explore SU experiences of using music technology in an MT intervention. (VII) To explore how music technology can be utilized in the subsequent analysis of audio data of musical interaction recorded during MT sessions.

We expect that an MT intervention, carried out alongside the ST, will (a) be feasible and acceptable for individuals receiving community-based substance misuse treatment; (b) lead to a decrease in the symptoms of anxiety, depression and craving that correlates with changes in the underlying neural processes; (c) improve treatment outcomes for SU in CSMTSs; (d) improve inhibitory control that correlates with a change in the underlying neural process and, potentially, with changes in the craving intensity.

### Study design

**Fig. 1:**
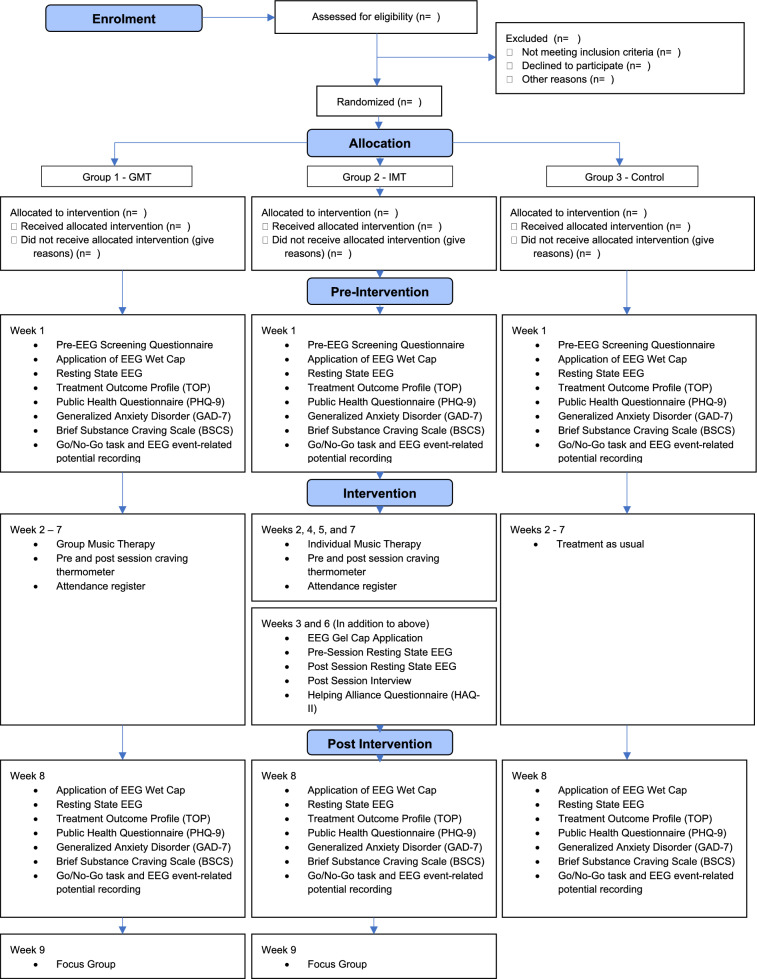
Consort flowchart representing study design and procedure

Westminster Drug Project (WDP) is a substance misuse service provider delivering services within the UK. WDP’s Innovation & Research Unit (IRU) and the Cambridge Institute for Music Therapy Research (CIMTR), Anglia Ruskin University, have designed a mixed-methods feasibility study to address these aims and objectives. The study is designed as a three-armed study (Fig. 1) with group 1 receiving 6 weekly sessions of GMT alongside ST (see section “interventions”), group 2 receiving 6 weekly sessions of IMT alongside ST, and group 3 serving as a control group (CG), receiving 6 weeks of ST only. All participants will take part in pretest–posttest assessment (i.e., baseline and post-intervention measures) including subjective (i.e., multi-item questionnaires), behavioral (i.e., Go/No-Go task) and EEG measurements to assess several mental-health-related clinical endpoints. SUs allocated to group 2 will receive IMT while simultaneously EEG-hyperscanning is being recorded during the 2^nd^ and 5^th^ therapy sessions. After these two sessions, participants will undergo a semi-structured interview to select moments of therapeutic interest and complete a multi-item questionnaire measuring the quality of therapeutic relationship. Each participant allocated to GMT and IMT will also complete a single-item questionnaire to assess the intensity level of craving. Feedback from participants will be collected throughout the study and, where possible, requests will be implemented into the subsequent session. Engagement and treatment status of participants will be assessed 6 months post-intervention. After the intervention two different focus groups will be conducted: one involving MT participants and another involving a Staff Group (SG) of 4–8 WDP staff members who work with the service users involved. The SG focus group will explore the feasibility of MT from several perspectives, including those involved in the set-up of the MT sessions. Whilst the duration of the entire study is outlined to be a period of 3 months, participants will be committed for 8 weeks.

### Patient and public involvement

The research team attended a SU consultation, organized by WDP, to present the project design and gather feedback from a SU perspective. A total of 7 SUs attended this consultation. The aim of this consultation was to get initial views of the design, participant time commitment, general interest in music therapy and thoughts on the EEG element. The research team received positive feedback from the SU's in attendance. The consultation also provided some input into the music therapy sessions, with suggestions on which instruments should be available. Further to this consultation, the research team will hold drop-in session within the WDP service during the recruitment window. This drop-in session will seek to aid recruitment and provide further information about the project and what participation will involve. Interested service users will also be able to ask the research team questions regarding the study and offer feedback.

### Participants

Fifteen SUs and, from a minimum of 4 to a maximum of 8 staff members, will be recruited from WDP. In total, we will recruit between 19 and 23 participants. The inclusion criteria for the recruitment of SUs include: (I) being a SU in structured treatment for drug and/or alcohol misuse, (II) being 18 years old or older, (III) reporting symptoms of depression and/or anxiety, (IV) being deemed suitable to take part in a group/individual setting by their key workers (who are Recovery Practitioners responsible for coordinating and delivering psychosocial interventions for SUs), (V) being right-handed.

The exclusion criteria for the recruitment of SUs include: (I) having a legal requirement to attend as part of a Drug Rehabilitation Requirement or Alcohol Treatment Requirement as this may impact engagement results, (II) being unable to provide informed consent due to a lack of mental capacity, (III) little/no understanding of spoken English as all sessions will be carried out in English and instructions for subjective measures used in this project are in English, (IV) being left- and mixed-handed: an outcome measure utilized (i.e., the EEG frontal alpha asymmetry) is modulated by handedness direction (i.e., left-handed, right-handed) providing empirical justification for the practice of excluding left-handers and mixed-handers when exploring relationships between this neural marker and other variables [34], (V) not being registered with a general practitioner (GP)/unable to provide GP details: we will require GP details to notify them in case of any incidental EEG findings. Furthermore, participants who disclose in the consent form that they had experienced a Stroke, Traumatic Brain Injury, or Epilepsy will be contacted by the research team to discuss if they can wear an EEG cap and participate in the measurements. Our inclusion and exclusion crit﻿eria are broadly based on the eligibility criteria for SUs to attend most groups in the service. We expect participants to either be abstinent or present with low to moderate substance use. Participants would also likely be housed and have access to public funds. While this presents a sample with diverse substance use patterns and individual circumstances, this is more representative of the cohort that would use this type of intervention. The diversity in participants may aid determining feasibility and acceptability of the intervention and provide value in planning further more focused studies. Regarding SG, only members of WDP staff will be included in this group.

### Recruitment

Opportunistic sampling will be used, with eligible SUs being identified and then provided an information sheet detailing the project. The service will also feature a poster advertising the project and asking interested SUs to contact WDP staff for more information. SUs volunteering to participate will be asked to complete an informed consent form. Once participants are recruited the researchers will randomly assign participants to one of the three groups (GMT, IMT or CG).

Recruitment will be carried out by advertising the project in the community and by WDP staff identifying eligible SUs through WDP's case management system. SUs can choose to opt out from being approached about research, so only those who have indicated that they are happy to be approached about research will be contacted. For those that have not been identified through the case management system but approached by their key worker, the key worker or service administrator will ascertain eligibility through the case management system and in keywork sessions. SUs identified as eligible will be provided with the information sheets and consent forms and asked to return these. Consent will be taken with a member of the research team or WDP staff present. This is to ensure that someone is there to help with questions and understanding. The long version of the participant information sheet is a comprehensive document including information on the project, ethical information, benefits, and potential risks of participating in the study. The short version of the participant information sheet includes a brief overview of the project and what participating in the study entails.

The staff cohort will be recruited by directly approaching staff members who were involved in the project or may be involved in future forms of the program. This will be done by emailing them directly or attending staff meetings and asking for volunteers to participate. Staff members will also be invited to attend the drop-in session. SUs who participate will be rewarded Capital Card^®^ points. Points will be awarded for each session completed, including the focus group and interview. WDP Capital Card^®^ is a reward card scheme to help SUs with their recovery journey ([35]; https://www.wdp.org.uk/the-capital-card). Where the project has issues recruiting the full number of 15 SU participants, we will extend the recruitment window to 2–3 months and we will re-run the drop-in session to attract more SUs. We will also contact WDP key workers to promote the study. Recruitment for GMT will continue until 5 participants are recruited and allocated to this group. If the 5 participants are not recruited within 2 months, the GMT sessions will start once at least 3 participants have been allocated. The GMT will continue while 3 participants remain in the study. The start of the IMT sessions will be staggered: SUs allocated to the IMT group will start their MT sessions within 1–2 weeks of being allocated to this group. If a participant in the IMT group drops out within the first 2 weeks, we will re-recruit and the new participant will start from week 1. We will only re-recruit twice before continuing with the remaining IMT participants. If after 3 months, the project does not have enough participants to go ahead, the research team will discuss additional recruitment strategies, widening the inclusion/exclusion criteria or a possible change in study location. The research team will take any feedback from SUs or staff around recruitment and incorporate where necessary. Where a participant decides to withdraw from the study, they will be provided an exit survey and withdrawal confirmation form. This form gives the participant the option to decide if the data collected up to their withdrawal will be kept and used in the final analysis or destroyed and not used in the final analysis. If they choose not to complete this survey or ask that all their data collected up to their withdrawal is deleted, the exit survey will be destroyed.

### Procedure

One week prior to the start of the interventions, the baseline measurement battery (secondary outcomes) will take place. Handedness will be assessed with the Edinburgh Handedness Inventory and a pre-EEG screening questionnaire will be administered. 

This includes questions about SU use of alcohol or any other substances, previous episodes of Stroke, Epilepsy, Brain Injury, or other conditions that may make wearing a cap or head garments difficult, uncomfortable, or painful. Furthermore, we will ask if participants are currently prescribed any antipsychotic, anti-depressive, or anti-anxiety medication. Quantitative and qualitative data will be collected through the outcome measuring tools (Table 1).

**Table 1:** Representation of primary and secondary outcomes, their labels and timepoints of administration

Outcome	Measure label	Measure timepoints
Primary outcome		
Feasibility and acceptability	Participant satisfaction survey	At Week 8
	Attendance sheet	After each session
	Exit questionnaire	Following disengagement with intervention
	Focus group	At Week 9
Secondary outcomes		
Substance use (a) Days of primary substance used (b) Usage of primary substance per day Physical Wellbeing Mental Wellbeing Overall Quality of Life	Treatment Outcome Profile (TOP)	Baseline and Week 8 (end of treatment)
Retention in treatment completion rate	WDP Case management system	At week 11
Substance craving	Brief Substance Craving Scale (BSCS)	Baseline and week 8 (end of treatment)
Neural correlates of substance craving	Beta frequency band/resting state	Baseline and week 8 (end of treatment)
Levels of depressive symptoms	Patient Health Questionnaire–9 (PHQ-9)	Baseline and week 8 (end of treatment)
Neural correlates of depressive symptoms	FAA/resting state	Baseline and week 8 (end of treatment)
Levels of anxiety	Generalised Anxiety Disorder–7 (GAD–7)	Baseline and week 8 (end of treatment)
Neural correlates of anxiety symptoms	FMT/resting state	Baseline and week 8 (end of treatment)
Inhibitory control	Visual behavioral Go/No-Go task	Baseline and week 8 (end of treatment)
Neural correlates of inhibitory control	EEG event-related potential (ERP) P3	Baseline and week 8 (end of treatment)
Therapeutic alliance	The Helping Alliance Questionnaire–II	After IMT session 2 and 5
Moments of therapeutic interest	Semi-structured Interview	After IMT session 2 and 5
	Audio-visual data	During IMT session 2 and 5

Individuals allocated to GMT and IMT will be asked to attend 6 weekly therapy sessions each with a duration of 60 min. However, due to the planned EEG hyperscanning and necessary preparation time (ca. 35 min) as well as semi-structured interviews (20 min) in weeks 2 and 5 in the IMT group, participants in this group are required to attend for ca. 115 min in these weeks. Individuals allocated to CG will be asked to attend their usual care by the WDP care team. The ST that participants will receive from WDP may vary from week to week but will include a range of psychosocial and clinical interventions delivered in 1:1 and group formats. Over the 6 weeks we expect a participant to receive up to 180 min of ST (approximately 3 × 60-min WDP key work sessions—see below) but this is depending on individual needs. The participant may also attend other groups or appointments at WDP. After the interventions, GMT, IMT and CG will complete the same secondary outcomes measures again. Primary outcomes will be measured after the interventions in weeks 8 and 9 with a participant satisfaction survey and focus groups, respectively.

### Interventions

MT involves the use of sounds and music within an evolving relationship between participants and therapist to support and encourage physical, mental, social, emotional, and spiritual wellbeing. Sessions will be delivered by a qualified music therapist, registered with the UK regulator for health and care professions, the Health and Care Professions Council (HCPC). Registered music therapists must have undergone training through an accredited Music Therapy training course, which is offered in the UK either in the form of a two year full time or three year part time masters level training (accredited by the HCPC).

MT sessions will be based upon musical improvisation, composing and writing songs, analyzing lyrics of preferred songs, singing and talking. Participants are not required to have any previous instrument or other music experience. During GMT, active and passive music activities will be conducted as a group. During MT sessions participants will be introduced to a range of both electronic and acoustic instruments, including tuned and untuned percussion, from which they can choose to play and improvise freely. The music therapist will accompany participants in the improvisation. This form of improvisation, with the support of the music therapists, provides participants with an opportunity to connect with emotions and feelings connected to unconscious material. Following improvisations, the therapist and SU may identify moments of interest which can be further explored either verbally or through further improvisation. Utilizing Electronic Music Production Instruments (EMPIs) provides participants with additional creative opportunities while utilizing contemporary timbres and music making techniques, thus offering participants a way to explore their musical and cultural identities [36]. The use of EMPIs also enables the recording of the music created in the sessions, which in turn provides dual benefits: firstly, participants can, if they wish, take a copy of the music they created away, and secondly, the recording can be used in the analysis of sessions.

GMT participants will have the opportunity to share any experiences and feelings arising with the group members. Through this shared experience group members can benefit from interpersonal learning, group cohesiveness and the development of social interaction techniques. ST includes 1:1 key work sessions, other group work programs and clinical support (e.g., prescribing, counseling, and clinical interventions).

### Outcome measures

Objective (i.e., EEG-based) and subjective (i.e., multi-, and single-item questionnaires), quantitative as well as qualitative (i.e., interviews and focus groups) measures will be used to accomplish the aims and objectives of the present study (Table 1).

### Outcome measures for each participant (GMT, IMT and CG)

In this section, we describe mental health, substance use and craving measures that will be administered prior to (baseline) and after (post-intervention) the intervention; therefore, at week 1 and week 8. To measure participants’ depressive symptoms, the Patient Health Questionnaire (PHQ-9) will be completed, and a resting-state EEG frontal alpha asymmetry (FAA) will be measured. The PHQ-9 is a validated and commonly used self-report to screen for depressive symptomatology [37] and shows a good internal consistency when administered to participants with SUD (Cronbach’s α = 0.90; [38]). The FAA is a neural biomarker representing the difference between the left and right alpha activity over the frontal regions of the brain [39] and a reduced FAA has been associated with depressive symptomatology [40, 41]. To measure participants’ anxiety symptoms, the Generalized Anxiety Disorder-7 (GAD-7) questionnaire will be completed and resting-state EEG frontal midline theta (FMT) will be measured. The GAD-7 is a validated [42] and standardized [43] self-report to screen for anxiety symptomatology showing a good internal consistency when completed by SUD participants (Cronbach’s α = 0.91; [43]). FMT is a neural biomarker that has been associated with anxiety [44] and increased resting-state FMT positively correlated with the reduction of anxiety after an MT intervention for clients with depression [16]. To measure participants’ feeling of craving in the preceding 24 h, the Brief Substance Craving Scale (BSCS) questionnaire will be completed and resting-state EEG beta frequency band will be measured. The BSCS is a validated questionnaire to assess craving in the SUD population over a period of 24 h [45]. The EEG beta frequency band is a neural EEG rhythm suggested to play a role in processing abstinence-related and cue-induced craving [46]; indeed, increased resting-state beta frequency band activity in the dorsal anterior cingulate cortex and pregenual anterior cingulate cortex have been associated with craving [46]. After the resting-state EEG recordings, participants will complete a behavioral Go/No-Go task while EEG event-related potentials (ERPs) will be measured to assess inhibitory control. The visual Go/No-Go task is a straightforward behavioral paradigm requiring participants to respond with a button press when a set of stimuli are presented (circles appearing at the top right and bottom left corners of the screen) and to withhold the response when another set of stimuli, with the same probability of occurrence, is presented (circles appearing at the top left and bottom right corners of the screen) [47–49]. To measure substance use, days of primary substance used, usage of primary substance per day, physical wellbeing, mental wellbeing and overall quality of life, the Treatment Outcome Profile (TOP) questionnaire will be completed. The TOP is a psychological health scale validated in a sample of SUD participants [50] and a standard measurement tool used in community treatments services in England and Wales. Biomarkers, such as Urine Drug Screening (UDS) or breathalyzers, will not be used during this feasibility study. This is due to costs and added burdens to the participants and the hosting service. While the TOP is a self-reported measure, it is a questionnaire regularly administered to all service users and we expect it to provide indicative results of which can be followed up in further studies.

### Additional outcome measures for participants in the GMT and IMT group

In this section, we describe outcome measures that will only be administered to participants assigned to the GMT and IMT group. Each participant and the therapist will complete an attendance sheet after each GMT and IMT session to monitor participation rates. To measure and monitor for instantaneous level of craving intensity, a Craving Thermometer (in the form of Visual Analogue Scale [VAS]) will be completed before and after each GMT and IMT session by each participant [51, 52]. This measure represents a quick-scan of craving intensity and a threshold score of 3 on a craving VAS ranging from 0 to 10 has been shown to sensitively predict the presence of craving (*p* < 0.05; [51, 52]). To assess if the MT program, carried out alongside the ST, is acceptable as an intervention in a community setting, participants will complete a participant satisfaction survey at week 8. Participants will also have the chance to provide detailed feedback on the MT program through the survey. To assess the feasibility and acceptability of MT in addition to ST in a CSMTS, a focus group will be conducted at week 9 (two weeks after the end of treatment) to collect qualitative data on SU satisfaction. An exit questionnaire will be administered to participants who disengage with the intervention to gather qualitative data regarding their reasons for withdrawing.

### Additional outcome measures for participants in the IMT group

In addition to the measures listed above, and to test the feasibility of investigating mechanisms of change with neurophysiological research methods, participants receiving IMT will undertake in-session measures. The following outcome measures will only be collected at the 2^nd^ and 5^th^ sessions. To measure client-therapist alliance, the Helping Alliance Questionnaire-II (HAQ-II) will be completed by the participant and the therapist [53]. The HAQ-II is a questionnaire measuring therapeutic alliance and has been validated in a large sample of SUD [53]. To select significant-nodal moments for the client, a qualitative semi-structured interview will be conducted after the session 2 and 5. Important and emotionally relevant moments of the 2nd and 5th therapy sessions can be examined by reviewing specific time frames acquired in the AV recording of the session and the corresponding EEG concomitants [22]. Additionally, during those two sessions, EEG hyperscanning technology will be adopted to measure the brain activity from the therapist and the participant while interacting. This method has already been implemented in an MT setting by members of the research team to explore the neural correlates of client-therapist relationship during selected therapeutically interesting moments [21, 22].

### Additional outcome measures for participants in the GMT group

Following completion of the MT intervention period, each GMT participant will be interviewed (audio recorded) by the music therapist. This individual qualitative debriefing interview will focus upon participant experiences of using music technology in music therapy sessions.

### Focus groups for participants and WDP staff

Following the completion of the entire intervention, the IMT, GMT and SG will be invited to attend focus groups lasting approximately 60 min. Two focus groups will be conducted. The first one for both the IMT and GMT participants. The second focus group will only include the SG. The focus groups will capture qualitative data on SUD participants’ acceptability, session feedback and suitability. The focus group for SG will explore feasibility and future implementation.

### Electroencephalography (EEG) recording

The pre-post intervention EEG recording procedure will include a 5-min eyes-closed resting-state, after which a 5.5-min EEG ERP recording during a Go/NoGo task will be performed. Those recordings will be accomplished utilizing 32 Ag/AgCl passive electrodes (R-Net, Brain Products, GmbH, Germany). The EEG resting-state frequency bands will be analyzed with Neuroguide (NG) software [16, 54, 55] while the ERP data will be analyzed using BrainVision Analyzer software (version 2.1.2; Brain Products GmbH) and MATLAB (R2021b) [56]. The EEG hyperscanning data will be recorded throughout the second and fifth therapy session from therapist and participant utilizing 32 Ag/AgCl active electrodes (ActiCap, Brain Products GmbH, Germany). Artifact control will be guided by video recording of the IMT session and by notes taken during recording. Pre-processing and processing steps to analyze neural time-series data will be performed with BrainVision Analyzer software (version 2.1.2; Brain Products GmbH) and will follow our previously published study [22]. All baseline resting-state EEG recordings will be reviewed for potential clinically relevant findings (incidental findings) by a Consultant in Clinical Neurophysiology (A.M.).

### Analysis strategy regarding the main study aim

Data analysis of the main study aim will be performed by comparing feasibility-related measures collected at the end of the treatment between participants allocated to the IMT or GMT groups. Descriptive statistics will be used to examine the rate of acceptability and satisfaction in these two experimental groups. Primary outcome measures (feasibility-related measures) that will be used are: (I) retention in treatment, which will be assessed by collecting attendance sheets and treatment status. The treatment status from participants receiving IMT, GMT and ST will be collected by WDP’s case management system 1-month post-intervention. This will provide information about retention in treatment, treatment engagement and completion rate. (II) Acceptability of treatment, which will be assessed by collecting a participant satisfaction survey as a post-intervention measure (at week 8) and implementing two post-intervention focus groups (FG1 and FG2) (at week 9). Primary outcome measures will only be administered/conducted after the end of the intervention with the aim of collecting the subjective perspective of the participants and staff involved in focus groups and will be subjected to thematic analysis using a specific thematic analysis model [57]. From this data themes will be identified pertaining (I) to participants' experience of MT in a CSMTS and (II) to participants’ experience of using music technology in music therapy.

### Analysis strategy regarding the secondary study aims

The strategy for the secondary aims entails data collection and analysis of quantitative and qualitative data from the EEG, questionnaires, a behavioral task, and a semi-structured interview. To analyze the effects of an MT program (GMT/IMT) in addition to ST compared to ST only on secondary dependent variables, descriptive, graphical, and inferential analyses will be used. Effect sizes will also be computed where relevant. The present research is a feasibility study and because of the small sample size, certain inferential statistics requirements cannot be satisfied. Therefore, results related to the secondary aims are to be considered—and will be presented as—preliminary. Descriptive analyses will be conducted by calculating means and standard deviations at each time point. Inferential statistical analyses will be conducted to evaluate the effect of MT (GMT/IMT) in addition to ST on mental health and brain processing by comparing the data between pre- and post- intervention in each group using a parametric or non-parametric test (e.g., paired t-test or Wilcoxon Signed-Rank test, respectively) depending upon the data distribution. The dependent variables that we will measure are pre-post intervention changes in the following outcomes: (I) score of the PHQ-9 and resting-state FAA for depressive symptoms; (II) score of the BSCS and resting-state beta frequency band for the craving state experienced over a 24-h period; (III) score of the GAD-7 and resting-state FMT for anxiety symptoms; (IV) score of the TOP for substance use, days of primary substance used, average usage of primary substance per day, physical wellbeing health, psychological health, and overall quality of life; and (V) P3 EEG event-related potential amplitude and behavioral accuracy during a Go/No-Go task for the inhibitory control. The statistical procedure to analyze data from pre-post intervention questionnaires involves both within-subject (or repeated-measures design) and between-groups (or independent design). Indeed, a pre-post intervention comparison of scores from the questionnaires will be performed for each group (GMT, IMT and ST) with a paired-samples t-test. In case the assumptions of normality and homogeneity of the data will be violated, a non-parametric Wilcoxon Signed-Rank test will be performed. The difference scores obtained from this repeated-measures design, will be used for the independent design analysis: pre-post intervention difference scores in the questionnaires administered to the MT groups (GMT and IMT) will be compared with pre-post intervention difference scores in the questionnaire administered to the ST group utilizing independent t-test. In case the assumptions of normality and homogeneity of the data will be violated, a non-parametric Mann–Whitney test will be performed.

For the resting-state EEG data (alpha—FAA -, beta, and theta—FMT—bands) pre-post intervention comparisons will be performed utilizing z-scores from a Normative EEG database (NG software) [54]. Furthermore, to represent the pre-post intervention difference in the topographic distribution of EEG power in the selected frequency bands a paired-samples t-test will be performed for each of the three groups. If deviations from normality will be found in the data, a non-parametric Wilcoxon Signed-Rank test will be performed. Between-group power differences will be calculated as well, by comparing differences in the topographic distribution of the EEG power in participants who received MT (GMT and IMT) alongside ST vs participants who received ST only through an independent t-test. If deviations from normality will be found in the data, a non-parametric Mann–Whitney test will be performed. Relationships between changes in the pre-post intervention questionnaire scores (PHQ-9, GAD 7, BSCS, VAS, HAQ-II) and changes in the respective EEG measures (see above) will be tested using Pearson's correlation coefficients with 95% confidence interval [16]. Likewise, we will implement the same correlation procedure to test the relationship between pre-post intervention changes in inhibitory control (P3 ERP amplitude and accuracy on “no-go” trials) and changes in the scores of the Brief Substance Craving Scale. Changes in the pre-post MT (GMT and IMT) sessions scores from the craving thermometer will be analyzed graphically (i.e., scatterplot). Those who attend more than two sessions but disengage before the study ends will have the measures up to the point of their disengagement included in data analysis.

### Analysis strategy regarding moments of therapeutic interest and music technology

A qualitative data analysis serves to transcribe, identify, and code relevant and pivotal moments of the therapy and associate them with neural markers of emotional processing, following the procedures described in previous studies conducted by members of the research team [21, 22]. These moments will be referred to as moments of interest (MOI) and will be selected and identified to describe a narrative of the therapeutic change process and to analyze EEG event-related temporal dynamics during MOI as compared to moments of non-interest (MONI). More specifically, we plan to analyze the time-course of EEG power in alpha and theta frequency bands [22], which have been shown to be associated with emotional processing and neuropsychiatric symptoms [40, 41, 44]. By examining changes in these frequency bands in therapist-client dyads across sessions, we may obtain an indication of the underlying neural mechanisms involved in the therapeutic process. Observations from the therapist and a semi-structured interview will be utilized to select MOI by importance and emotional relevance. Participants may also report verbally any craving-related experience, which would also allow us to describe instances of neural dynamics of craving. MOI will be identified and located in the AV recordings of sessions. These reports and their timestamp of occurrence will be entered into a therapy event log. This semi-structured interview will also be conducted to explore participants' perspective and experience of using music technology in a music therapy intervention. HAQ-II will be completed by the IMT group and by the therapist after session 2 and 5 to graphically explore the dynamics of the therapeutic relationship and correlate with outcome measures. Audio–video recordings of individual sessions will be transcribed with ELAN [58] Version 6.2, Max Planck Institute for Psycholinguistics, Nijmegen, The Netherlands). MOI and MONI will be identified by the client, the music therapist and by an independent music therapist acting as a second rater. The analysis of interview responses and transcriptions of video recordings will be subjected to thematic analysis [57]. From this data themes will be identified pertaining (I) to participants' experience of MT in a CSMTS and (II) to participants’ experience of using music technology in MT. The research team will identify themes within the free text boxes of the post-MT questionnaires, satisfaction and exit surveys, and the discussions recorded within the focus groups. Audio recordings of the musical components will be analyzed using aural and spectral analysis while Musical Instrument Digital Interface (MIDI) data will be subject to computational analysis using the Music Therapy Toolbox (MTTB) [33].

## Discussion

### Expanding the music therapy research evidence base into community settings

The main reason to conduct this study is to report on the feasibility of implementing an MT intervention for SUD clients engaged with a CSMTS (five receiving IMT and five receiving GMT). Whilst the efficacy of MT for SUD has been suggested by previous research, there is a gap in the research literature concerning SUs engaged with CSMTSs [4, 6, 59]. This may be due to several reasons but – reflecting on our (DM, DDS) experience of working in community settings  CSMTSs may face unique challenges that are not often experienced in residential or private treatment centers. Some of the factors that can differ between interventions in CSMTSs and private/residential treatment services, include (I) SUs may have no contact with each other in-between sessions; (II) greater length between sessions; (III) larger opportunity for negative experiences between sessions; (IV) uncontrolled environment before and after sessions; (V) the commissioning process of substance misuse services can result in a change of quality and type of care where new providers are commissioned to provide the service; (VI) larger turn-over of staff; (VII) frontline staff with a higher caseload can result in less 1 to 1 time. However, it is worth highlighting some of the advantages of community over inpatient treatment: the main reason for community-based treatment is that it is open access (unlike inpatient, where the individual or local authority has to apply for and fund their stay). An additional advantage of delivering interventions in the community is it allows SUs to benefit from the effects of the intervention, techniques (such as managing triggers and cravings) and changes within their everyday environment. Delivering treatment within a community setting therefore removes one of the main risks of inpatient settings: discharge back to an environment which may be triggering and without techniques that have been tried and tested within such environments. They are receiving treatment in their natural environment which means they are putting skills such as managing triggers and cravings, into practice earlier on. Music therapy, we hope, will be another such support mechanism and given the current evidence base will assist to reduce craving and increase engagement in SUs [4].

### Craving and mental health

With the present study, we expect to report positive results regarding the feasibility of MT in CSMTSs that could benefit society through the development of MT intervention in community settings for SUD. This is especially important since, to date, there is no specific psychotherapeutic or pharmacological treatment to improve the mental health and reduce relapses in people with SUD demands [12, 25]. Results from this study may also help to provide more insight into outcomes related to co-occurring mental illness, a research target area set in a 2021 issued independent review from the UK’s Department of Health and Social Care [60]. Furthermore, primary results regarding the feasibility of MT for CSMTSs will influence the development of larger studies with appropriate, acceptable, and practical research procedures. As secondary purposes, this mixed-methods randomized controlled feasibility study aims at implementing a pretest–posttest methodology comparing MT in addition to ST vs ST alone on neurophysiological (EEG assessment) and questionnaire-based measures to provide novel and preliminary information concerning the effect of an MT intervention on craving and mental health-related clinical outcomes. In addition, qualitative measures regarding the therapeutic relationship as well as the application of music technology within MT will be explored. Therefore, the study will contribute to the existing literature regarding the feasibility of adopting such a mixed methodology in a CSMTS.

### Sample size and dosage

Secondary purposes will be investigated within the frames of a feasibility study that include, as a corollary, the recruitment of a relatively small sample size to have preliminary data to determine whether interventions and research procedures are appropriate, acceptable, and practical [61].

Therefore, conclusions regarding mental health clinical endpoints as well as the therapeutic relationship and the role of music technology will be interpreted with care and presented as preliminary. Presently, positive results regarding the effectiveness of MT, as compared to a ST provided by the community, reflecting an improvement in the mental health of SUD participants have not been reported yet; for this reason, notwithstanding the small sample size limitation, it is essential to fill this gap in the literature. Additionally, analysis of the primary and secondary study aims will be performed on an intention-to-treat basis to manage drop-outs and missing data. A total of 6 IMT and GMT sessions will be offered. Patients with fewer sessions will not be excluded from data analysis (intention-to-treat principle; [62]). Nevertheless, to minimize missing data imputation, for participants not attending the post-treatment test battery at week 8, the last observation carried forward (i.e., in this case, baseline measurements at week 1) will be used.

Whilst depressive [26, 27], anxiety [26, 28, 29] and craving [30–32] symptoms have been shown to improve after MT in individuals with SUD, this will be a first attempt to study these dimensions in a community setting by measuring their neural correlates.

### Neural correlates and corresponding outcomes related to craving and mental health

Specifically, we expect to provide preliminary positive results regarding a reduction in depressive symptomatology after an MT intervention, as indicated by lower scores on the Patient Health Questionnaire—9 [37] and increased resting-state FAA (higher left-sided frontally distributed power in alpha range [40, 41]); a reduction in anxiety symptomatology after an MT intervention, as indicated by lower scores on the Generalized Anxiety Disorder assessment—7 [42] and increased resting-state FMT [16, 44] and a reduction in short-term craving symptomatology after an MT intervention, as indicated by lower scores at the Brief Substance Craving Scale [45] and reduced power in the beta frequency band measured at rest [46]. The neural correlates of depressive, anxiety and craving symptomatology will be measured by means of resting-state EEG to investigate the intrinsic activity [63] of the brain and utilizing neurometric comparisons against a normative EEG database [16, 54]. In this way, we can examine the relationship between pre-post intervention changes in the multi-item questionnaires scores and pre-post intervention changes in the neural activity that is hypothesized to underpin depressive, anxiety and craving symptomatology. Since the experience of craving is related to a lack of control over intrusive drug-related thoughts [64, 65] and lack of behavioral control [66, 67], it can be approached with behavioral tasks requiring the deployment of inhibitory control functions (e.g., response inhibition) [11, 64]. The effect of MT on inhibitory control will be assessed using a behavioral task (Go/No-Go task), changes in the EEG event-related potential P3 and by measuring the correlation between inhibitory-related measures and short-term craving measures. It is expected to provide preliminary positive results regarding an improvement in inhibitory control after an MT intervention, as indicated by a higher accuracy in the Go/No-Go task and increased NoGo P3 event-related potential [47–49]. Furthermore, we expect a negative correlation between inhibition indexes (increased pre-post intervention NoGo-P3 brainwaves and higher pre-post intervention accuracy on NoGo trials) and scores in the BSCS. In other words, it is expected that an improvement in inhibitory control skills after an MT intervention would be associated with decreased subjective feeling of craving. A second measure to assess craving will be used. Since music represents an environmental cue, it can be associated with the drug experience and, potentially, induce craving by triggering emotional memories [68, 69]. Therefore, to monitor for instances of addiction memories that can possibly induce craving [15], an instantaneous single-item measure of craving will be collected before and after group and individual music therapy sessions. While MT has been suggested to reduce craving [30–32], the element of music itself, in some circumstances, can determine the induction of a craving state [68, 69] and there is no systematic empirical evidence about the mechanisms underlying the rising of moments of craving in MT sessions [4].

### Process related outcomes and analysis

For this reason, we have been implementing a close monitoring for instances of addiction memories and potential peaks of craving to study if, how and when these instances occur. Working with a qualified music therapist, individuals can begin to experience non-drug induced emotions through the exploration and re-creation of preferred music pieces that were originally linked to drug-induced emotional experiences and opening up new pathways of emotional exploration [70, 71]. This treatment perspective, through retraining and reframing emotional intensity, builds on gradually decreasing and recalibrating the emotional impact related to music-induced memories [15]. Moments of state-dependent recall, during which individuals may experience cravings or urges relating to their drug use offer music therapists an opportunity to work with individuals towards retraining and reframing musical cues. Understanding how the therapist recognizes these moments can provide important insight into the therapeutic process. To investigate this, we will administer a craving thermometer pre-post MT session, a post MT session debriefing with the therapist and a more intense feasibility investigation, including a post-session questionnaire (HAQ-II) and dual-EEG with video session monitoring (to revisit instances) during IMT sessions 2 and 5. With EEG hyperscanning and HAQ-II we aim to monitor and investigate the neural dynamics of emotional processing deploying within the MT process with the use of music technology. During these two IMT sessions, we will test the feasibility of using EEG hyperscanning and AV monitoring to examine the neural correlates of therapeutically relevant moments involving craving, intense emotion, and therapeutic interpersonal interaction, and we expect that these moments will occur more frequently in the later sessions. This research approach has already been shown to provide valuable insights into the neural dynamics of shared emotional processing during a music therapy session [22]. By segmenting the EEG data corresponding to therapeutically important moments of the audio–video recorded therapy session (such as moments regarding craving and intense emotions, as determined from post-session interviews with participants and by observations from the therapist), we plan to analyze and contrast these segments with moments not selected as pivotal or emotionally relevant (i.e., moments of non-interest). Furthermore, we will record and analyze participants’ engagement with electronic music production instruments (EMPI) and acoustic instruments. With this methodological approach, we can explore facets of therapeutic alliance that is considered a non-specific factor and a central element of any kind of psychotherapy, MT, and art therapy [72, 73] by analyzing temporal dynamics of contextualized events and therapeutically relevant moments [21, 22].

## Conclusion

With this feasibility study we aim to lay the groundwork for researching MT interventions in CSMTSs, addressing a gap of research into mechanisms of change regarding craving and related mental health outcomes. We propose a mixed-methods approach created with feedback from service users and practitioners addressing clinical practice and research needs. The procedures have been adapted to clinical practice and with this small-N feasibility study we aim to create an empirical base for a larger-N RCT drawing on ‘different disciplines and bring[ing] practitioners and researchers together’ to find out ‘what works to combat substance misuse, across supply, prevention, treatment and recovery’ [60].

## Data Availability

Not Applicable.

## Abbreviations

AV: Audio–video
BSCS: Brief Substance Craving Scale
CIMTR: Cambridge Institute for Music Therapy Research
CSMTS: Community Substance Misuse Treatment Service
CG: Control group
EHI: Edinburgh Handedness Inventory
EEG: Electroencephalography
EMPI: Electronic Music Production Instruments
ERP: Event-related potentials
FG: Focus group
FAA: Frontal Alpha Asymmetry
FMT: Frontal Midline Theta
GP: General practitioner
GAD-7: Generalized Anxiety Disorder-7
GMT: Group music therapy
HAQ-II: Helping Alliance Questionnaire-II
IMT: Individual music therapy
IRU: Innovation & Research Unit
IRAS: Integrated Research Application System
MOI: Moments of Interest
MONI: Moments of non-Interest
MT: Music therapy
MTTB: Music Therapy Toolbox
MIDI: Musical Instrument Digital Interface
PHQ-9: Patient Health Questionnaire-9
SU: Service user
SG: Staff group
ST: Standard treatment
SUD: Substance Use Disorder
TOP: Treatment Outcome Profile
VAS: Visual Analogue Scale
WDP: Westminster Drug Project
